# The Treatment of Gastroptosis

**Published:** 1902-03

**Authors:** 


### The Treatment of Gastroptosis.

In discussing the treatment of gastroptosis in the February number of the *Pennsylvania Medical Journal,* Dr. J. Dutton Steele expresses the belief that the character of food allowed should be determined by the amount of hydrochloric acid present. An exclusive milk diet is not usually well borne. Only small amounts of food should be taken at a time and eating should always be followed by a period of rest. The stomach must be held in place by an abdominal belt or binder, the lower edge of which should be held against the pubes by perineal bands. The binder must be supplemented by the use of pads so placed that they will exert upward pressure on the stomach. With the use of a proper binder and pads relief follows almost instantly. This treatment allows the stomach to re-establish its compensation. R.

